# Lemierre’s Syndrome

**DOI:** 10.31662/jmaj.2025-0207

**Published:** 2025-09-05

**Authors:** Kiyozumi Suzuki, Hiromasa Otsuka

**Affiliations:** 1 Department of General Medicine, Ageo Central General Hospital, Kashiwaza, Ageo-shi, Saitama, Japan

**Keywords:** Lemierre’s syndrome, *Fusobacterium necrophorum*, septic thrombophlebitis, septic emboli

A previously healthy 23-year-old man presented to a local clinic with a 1-week history of fever and sore throat, which progressed to cough, hemoptysis, chest pain, and dyspnea. The patient was in shock and experiencing respiratory failure. A chest radiograph revealed multiple pulmonary masses (Figure 1A), raising suspicion for metastatic lung cancer, and prompting transfer to our hospital.

**Figure 1.**
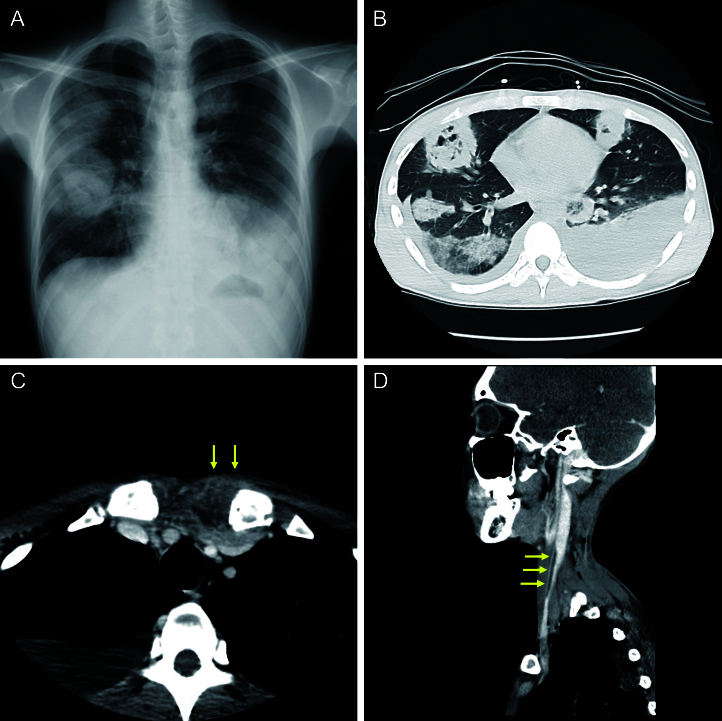
(A) Chest radiograph revealing multiple large, rounded opacities in both lung fields. (B) Chest computed tomography (CT) revealing multiple bilateral pulmonary cavities and bilateral pleural effusions. (C) Contrast-enhanced CT revealing increased thickness of the soft tissue around the left sternoclavicular joint (arrows) and (D) a filling defect in the right internal jugular vein (arrows).

On arrival, laboratory data revealed elevated inflammatory markers, multiorgan dysfunction, and disseminated intravascular coagulation. Computed tomography showed numerous cavitating pulmonary lesions suggestive of septic emboli, empyema (Figure 1B), sternoclavicular arthritis (Figure 1C), and a thrombus in the right internal jugular vein (Figure 1D). Blood cultures yielded growth of *Fusobacterium necrophorum*, susceptible to most β-lactam antibiotics and clindamycin. These findings confirmed the diagnosis of Lemierre’s syndrome (LS). The patient was administered meropenem and underwent chest drainage. After significant clinical improvement, antibiotic therapy was switched to oral amoxicillin-clavulanic acid, completing a full 3-month antibiotic course, which resulted in full recovery.

LS is a rare but life-threatening condition that primarily affects otherwise healthy young adults and is characterized by an antecedent oropharyngeal infection, *Fusobacterium* septicemia, thrombophlebitis, and metastatic infections ^(1)^. The lungs are the most commonly affected site of metastatic infection in LS, often presenting with bilateral necrotic pulmonary emboli, pleural effusions, empyema, or lung abscesses ^(1)^.

Although imaging studies are key diagnostic tools for LS, early diagnosis and appropriate therapy require a thorough understanding of this distinctive condition. Clinicians encountering oropharyngeal infections in routine clinical practice should be familiar with the clinical features of this potentially fatal disease.

## Article Information

### Author Contributions

Kiyozumi Suzuki: Writing - Original draft, Methodology. Hiromasa Otsuka: Methodology, Writing - review & editing. All authors critically reviewed the manuscript.

### Conflicts of Interest

None

### IRB Approval Code and Name of the Institution

In this study, Institutional Review Board approval was not required. Consent was obtained from the patient for the use of images for publication.
